# “I make efforts, people make comments”: Prof. H. Zanyin Gaw—pioneering the world, the trailblazer and founder of China’s virology research

**DOI:** 10.1007/s13238-015-0227-4

**Published:** 2015-11-07

**Authors:** Huan Liu, Han Zhang, Doudou Chen

**Affiliations:** Wuhan Institute of Virology, Chinese Academy of Sciences, Wuhan, 430071 China; State Key Laboratory of Virology, Wuhan, China

**Figure 1:**
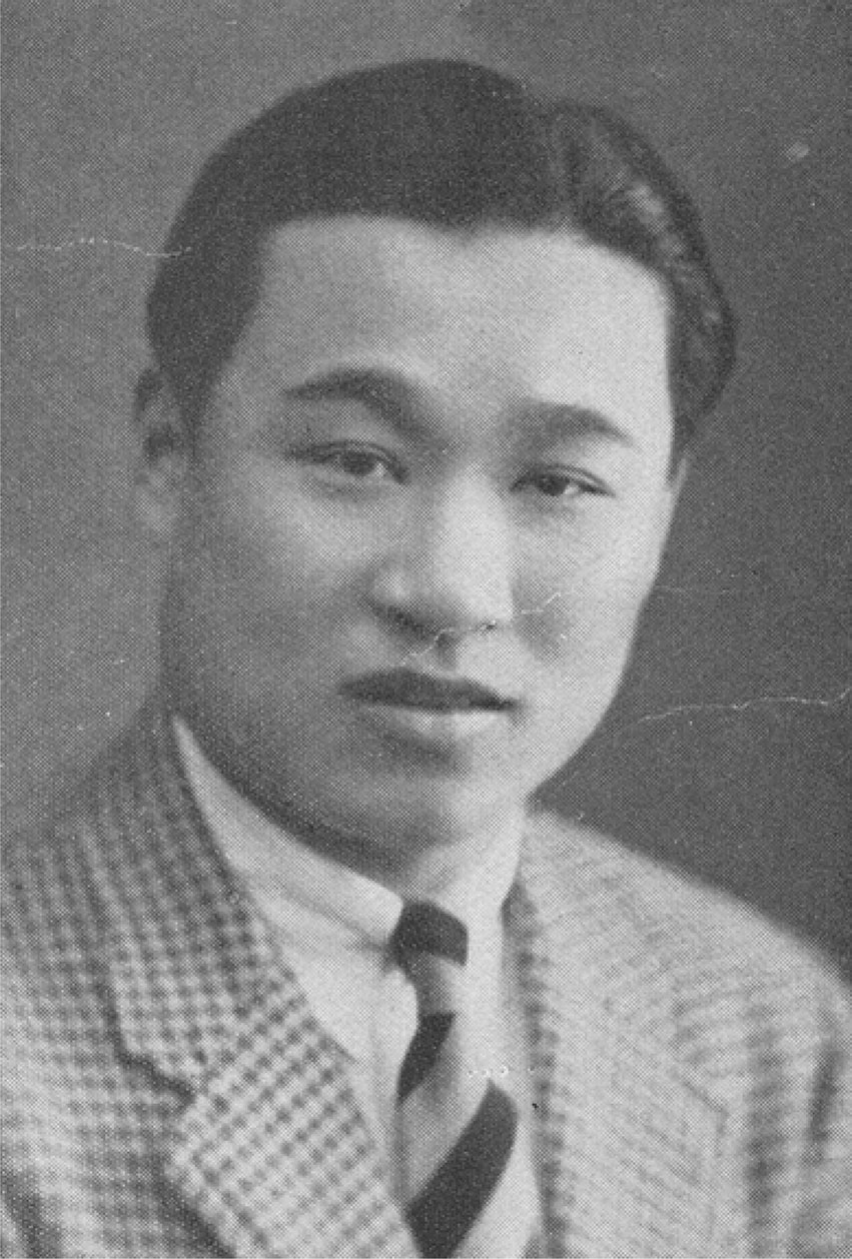
**Prof. H. Zanyin Gaw**

**Figure 2:**
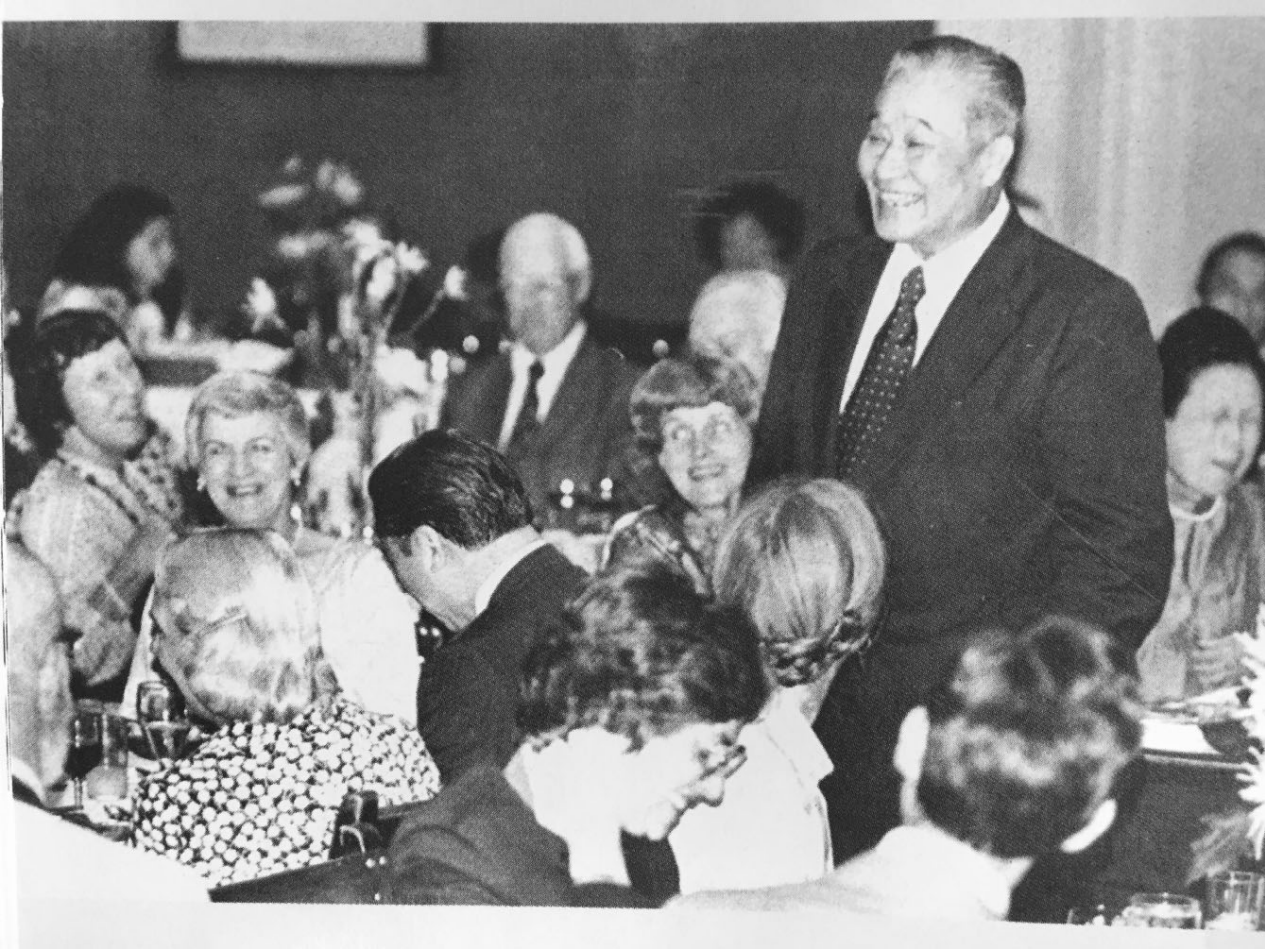
**Prof. H. Zanyin Gaw at the Yale-China Association**

“As a scientist and educator, Professor Harry Zanyin Gaw (H. Zanyin Gaw) enjoyed an outstanding reputation both at home and abroad. For more than half a century, he was always erudite and studious, devoting himself to the advancement of science and education of China, always keeping pace with global development trend of biology.” Prof. Minyou Qi, the former president of Wuhan University appraised (Hu et al., 2002; Zanyin Gaw, 2007) . Prof. Gaw was one of the founders of virology in China. He established the first virology research institute, the first microbiology major and the first virology major of China. In 1958, his research on the silkworm pus virus tissue culture represented a breakthrough of invertebrate tissue culture and insect virus research. His book *Theory and Applied Research on Insect Viruses* was received to worldwide widespread acclaim. In 1980, he was elected as a member of the Academic Divisions of the Chinese Academy of Sciences (CAS). Throughout his 56 years of research and education, he made tremendous contributions to the progress of Chinese microbiology and virology (Figs. 1 and 2).

Prof. Gaw was born on March 3rd, 1909 in a family of scholars of Taozhuang Township, Jiashan County, Zhejiang Province. In 1926, majoring in biology, he became a college student of Dongwu University. In 1930, he was awarded a Bachelor of Science Degree. When he was 21 years old, he was offered a scholarship by Lawrence University and obtained a Bachelor of Letters in only one year. In 1931, he began his postgraduate education in Yale University. His dissertation *“Physiology of the Contractile Vacuole in Ciliates”* won praise from his tutor and experts, and in early 1935, Prof. Gaw was awarded a doctor’s degree. Though being invited to stay in the United States, he was concerned more about his impoverished motherland and its lack of virology knowledge and committed himself to returning home.

In August 1935, Prof. Gaw came back to China and at the age of 26 he became the youngest professor of Wuhan University. Between 1935 and 1945, he taught general biology, microbiology, and other courses at Wuhan University. In 1945, as a visiting researcher, Prof. Gaw went to the Rockefeller Institute for Medical Research and engaged in virology research in the laboratory of the Nobel Prize Laureate W. M. Stanley. In 1947, after declining an offer of Rockefeller Institute and the invitation of the Royal Society of Britain, he returned China and founded the first virology research laboratory of China. In 1955, led by Prof. Gaw, Wuhan University established the first microbiology major domestically (Hu, 2004).

In January 1956, Prof. Gaw answered the Call to “March towards Science”. He took the initiative to establish the Wuhan Institute of Virology, CAS. He worked with Prof. Huagui Chen and other scientists to establish the Wuhan Microbiological Research Preparatory Committee, later renamed to the Wuhan Institute of Virology in 1978, which was the first research institute of virology in China. The institute was dedicated to basic virology research and was aimed at serving the demands of industry, agriculture and national defense. Prof. Gaw provided outstanding leadership for the institute for 28 years, and enabled the institute to make significant progresses and several groundbreaking discoveries in China.

While working in the lab of W. M. Stanley, Prof. Gaw published several influential academic papers including a paper entitled: ‘*A Comparative Study of the Properties of Two Strains of Tobacco Mosaic Virus Prepared from the Sap and from the Leaf Residues of Diseases Turkish Tobacco Plants*’, where he described the stability of physicochemical properties of virus and proved that the virus’ properties, especially its physicochemical properties remained the same despite separate hosts. This research result, which was highly appraised by peer scientists, has been widely referenced in professional literatures throughout the world. After returning from the U.S., Prof. Gaw continued to develop this research in Wuhan University, proving the physicochemical properties of influenza virus’ successfully (Zanyin Gaw, 1953), despite the difficulties in experimental budgets and research conditions.

In 1957, he led a research team to commit to *Research on Cultivation of Virus of Grasserie in Silkworm Tissue Cultures*, and presented the thesis of *Culturing all types of Silkworm Tissues Using Monolayer Culture* at the 6th International Invertebrate Virology Conference in Czechoslovakia in 1958, which aroused a strong response among the attending experts. The use of the monolayer tissue culture method for research into silkworm pus disease virus by Prof. Gaw and his research team represented a major breakthrough in invertebrate tissue culture and insect viruses research.

Outbreaks of silkworm infectious diseases could cause severe loses in silk production. Compared with feeding of silkworms, tissue culture is season-independent for virus propagation; the pus virus was considered to be proliferated only in ovaries. Prof. Gaw and his colleagues were able to culture the monolayers cells obtained from male and female gonads, trachea, muscle, intestine and silk-gland tissue. To prepare monolayer cultures, they used two methods: cell suspension culture and cells obtained by trypsinization. Ultimately, they succeeded in maintaining subcultures of male and female gonad cells for twenty-two generations, which can maintain a normal phenotype and represent the typical cytopathic effect after virus infection. This elegant technique, permitting continuous studies of the virus disease without raising silkworms, is now regarded by international virologists as a pioneering work in this field.

Prof. Gaw always quoted Karl Marx, who once said that “*Science must not be a selfish pleasure*. *Those who have the good fortune to be able to devote themselves to scientific pursuits must be first to place their knowledge at the service of humanity*”, to inspire students. During his scientific research, he came across difficulties and frustrations, but Prof. Gaw always had unswerving dedication and stood on the leading edge of advanced biological science. Making significant achievements for China, Prof. Gaw always said “*I Make Efforts*, *People Make Comments*”. Prof. Gaw has left a valuable and generous spiritual fortune for us. His entrepreneurship of “Pioneering the World” will continue to lead youth to march towards the advancement of science, the lessons from Prof. Gaw should be everlastingly cherished and never forgotten.
